# Concomitant inhibition of receptor tyrosine kinases and downstream AKT synergistically inhibited growth of KRAS/BRAF mutant colorectal cancer cells

**DOI:** 10.18632/oncotarget.14009

**Published:** 2016-12-17

**Authors:** Qiaoling Song, Xiaoxiao Sun, Hui Guo, Qiang Yu

**Affiliations:** ^1^ Shanghai Institute of Materia Medica, Chinese Academy of Sciences, 201203 Shanghai, China; ^2^ University of Chinese Academy of Sciences, 100049 Beijing, China

**Keywords:** receptor tyrosine kinases, AKT, colorectal cancer, drug combination, RAS/RAF

## Abstract

Receptor tyrosine kinase (RTK) signaling pathways are frequently activated in cancer cells due to mutations of RTKs and/or their downstream signaling proteins such as KRAS and BRAF. About 40% colorectal cancers (CRCs) contain KRAS or BRAF mutant genes and are resistant to treatments with individual inhibitors of RTKs, AKT, MEK, or BRAF. Therefore, an understanding of the molecular mechanisms of the drug resistance is necessary for developing effective strategies to treat the diseases. Here we report the discovery of an AKT/ERK reactivation mechanism that account for the cancer cell resistance to the AKT and MEK inhibitors treatments. The reactivations of AKT and ERK after the AKT or MEK inhibitor treatment were caused by a relief of an AKT or ERK-mediated feedback inhibition of the RTKs and/or their downstream pathways. A combination of RTK inhibitors, based on the RTK activation/phosphorylation profile, synergized with the AKT inhibitor, but not the MEK inhibitor, to completely inhibit the AKT phosphorylation and to block the growth of KRAS/BRAF mutant CRC cells. These results underscored the importance of AKT and the AKT feedback signaling to cancer cell growth and offered a novel therapeutic approach for the treatment of KRAS/BRAF mutant CRC cells.

## INTRODUCTION

Receptor tyrosine kinases (RTKs) signaling pathways are the major pathways in regulating cell proliferation, differentiation, and survival [1, 2]. The RTKs are activated by growth factors and transduce signals through two distinct downstream pathways: the mitogen-activated protein kinase kinase (MEK)/extracellular signaling-regulated kinase (ERK) pathway and the phosphatidylinositol 3-kinase (PI3K)/protein kinase B (AKT) pathway [3–5]. These two pathways are frequently activated in cancer cells due to aberrant activation of RTKs and/or activating mutations in their downstream signaling molecules such as KRAS and BRAF [6–9]. Specific RTK inhibitors (RTKis) have been used for the treatment of certain cancers with RTK activations [10–12]. However, therapeutic efficacies are low in most cancers with KRAS or BRAF mutations [13, 14]. Although inhibitors of the downstream effectors such as RAF and MEK exhibit good therapeutic efficacies in BRAF mutant melanoma cells [9, 15], most cancer cells are resistant to the single inhibitor treatments [16–20].

Colorectal cancer (CRC) is the third most common cancer and the fourth leading cause of cancer deaths worldwide [21–23]. So far, there have been only two types of targeted therapies approved for CRC treatments, an anti-angiogenesis therapy and an anti-EGFR therapy. Cetuximab and panitumumab, two antibodies against EGFR, can only prolong survival of colorectal cancer patients with normal KRAS [24]. However, the data in the Catalogue of Somatic Mutations in Cancer (COSMIC) indicate that 41.6% (2463 of 5926) of the CRC patients contain KRAS or BRAF mutant genes [25, 26], suggesting that a large population of CRC patients can not profit from the anti-EGFR treatment and new therapeutic strategies are urgently needed.

In this study, we investigated the drug resistance mechanisms of the KRAS or BRAF mutant CRC cells and explored new strategies to target the RTK signaling pathways for the treatment of the CRC cells. We found that AKT inhibitors (AKTi) and MEK inhibitors (MEKi) individually or in combination were not sufficient to inhibit the growth of the KRAS/BRAF mutant CRC cells. A reactivation of AKT or ERK, which was caused by a relief of a feedback inhibition of the RTKs and/or their downstream signaling pathways, occurred after the inhibitors treatments. An RTKi combination, based on the RTK phosphorylation profile in the cancer cells, synergized with AKTi, but not MEKi, to inhibit the reactivation of AKT and the growth of the CRC cells. Our findings underscored the key role of the RTK-PI3K-AKT pathway in the KRAS/BRAF mutant CRC cells, and provided a novel strategy for CRC treatment.

## RESULTS

### AKTi and MEKi were insufficient to inhibit growth of most of the KRAS or BRAF mutant CRC cells

**Figure 1:**
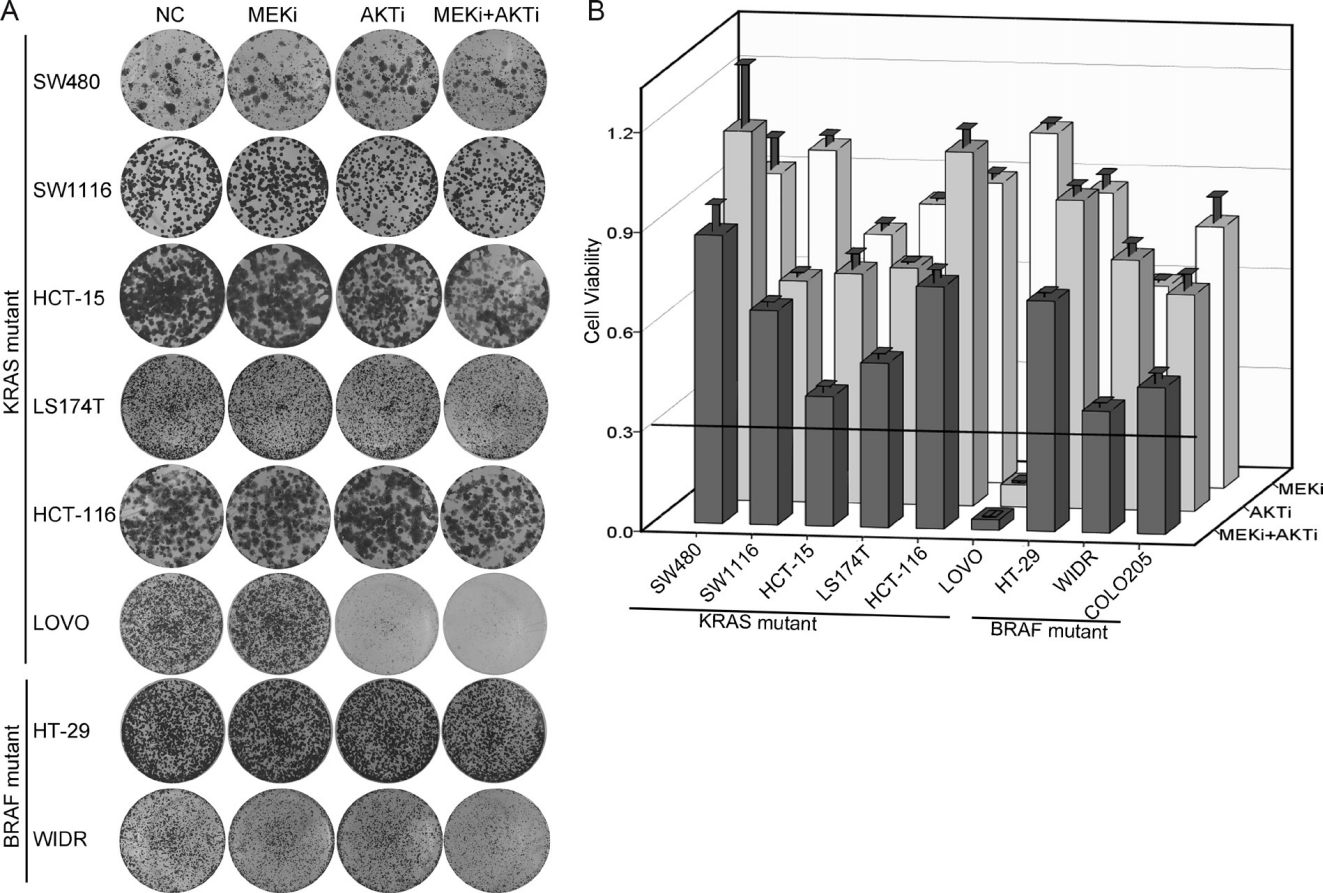
AKTi and MEKi were insufficient to inhibit growth of most of the KRAS/BRAF mutant CRC cells (**A**) Foci formation assay of six KRAS mutant CRC cell lines (SW480, SW1116, HCT-15, LS174T, HCT-116 and LOVO) and two BRAF mutant CRC cell lines (HT-29 and WIDR). Cells were treated with DMSO (NC), 0.25 μM MK2206 (AKTi), or 2.5 μM U0126 (MEKi) individually or in combination and visualized by crystal violet staining at the endpoint. (**B**) Quantification of the crystal violet staining in (A). For COLO205 cells, absolute cell numbers were counted by MUSE cell analyzer. Relative cell viability was calculated by comparing to the vehicle treatment. A cutoff of relative cell viability at 0.3 was drawn to define drug-sensitive and drug-resistant effects. The data was graphically represented as mean ± SD.

We collected nine KRAS or BRAF mutant CRC cell lines and inhibited the MEK/ERK pathway and/or the PI3K/AKT pathway downstream of RAS-RAF with a MEK inhibitor (MEKi) U0126 and/or an AKT inhibitor (AKTi) MK2206 [27, 28]. The concentrations of the two inhibitors we used were sufficient to completely inhibit the phosphorylation of MEK and AKT *in vitro*. The foci formation experiments demonstrated that for most cells, inhibition of both MEK and AKT led to less than 70% cell growth inhibition (13% for SW480, 36% for SW1116, 61% for HCT-15, 51% for LS174T, 27% for HCT-116, 31% for HT-29, 63% for WIDR and 56% for COLO205) (Figure 1A and 1B). LOVO cell line was the only cell line that was dramatically inhibited by the AKTi (93% growth inhibition) and by the combination of the AKTi and the MEKi (97% growth inhibition). These data indicated that the AKTi and MEKi were insufficient to inhibit the growth of most of the KRAS/BRAF mutant CRC cells.

### AKT and ERK were reactivated after the AKTi and/or MEKi treatments

**Figure 2:**
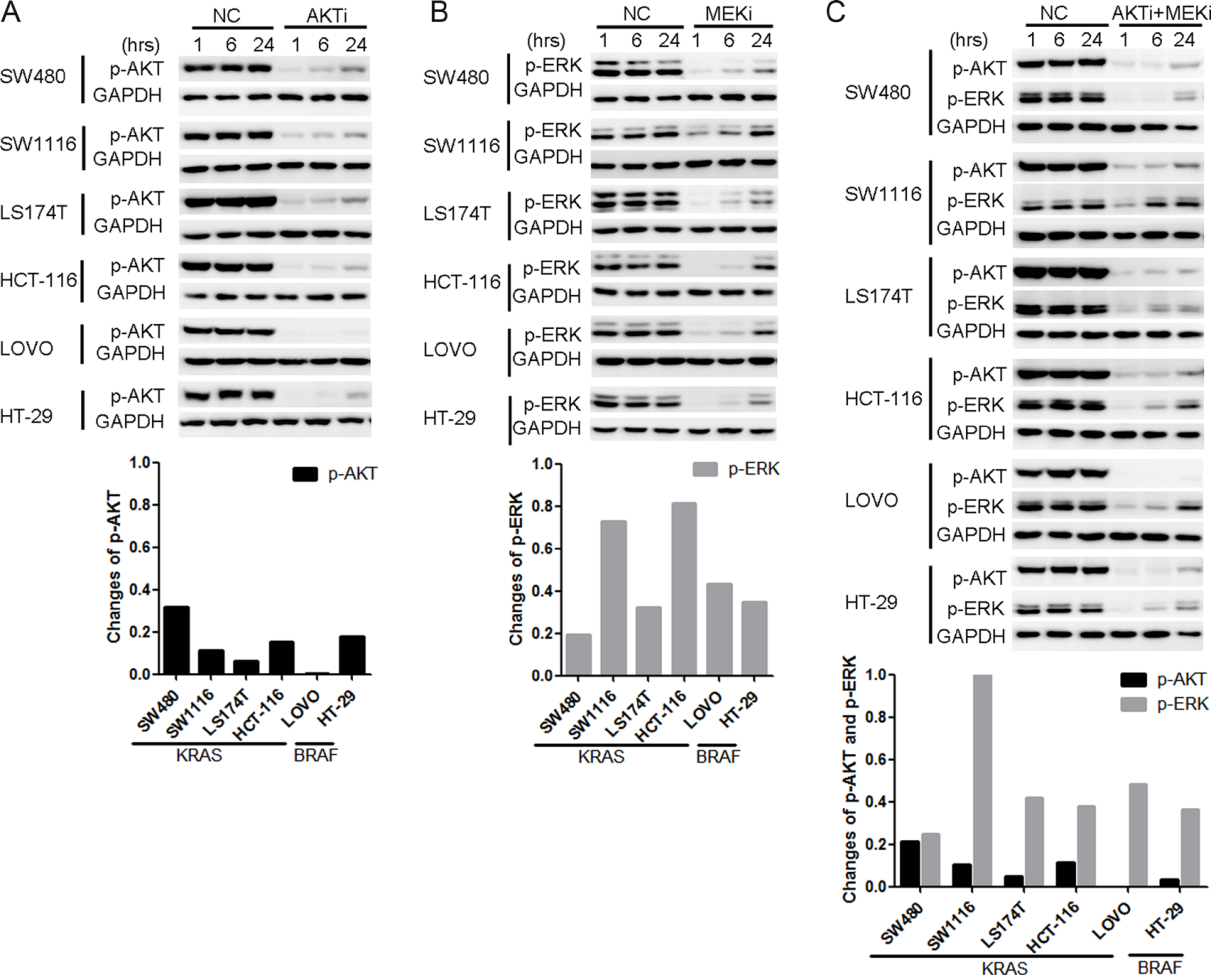
AKT and ERK were reactivated after the AKTi or/and MEKi treatments (**A**–**C**) Cells were treated with DMSO (NC), 0.25 μM AKTi (A), 2.5 μM MEKi (B), or the combination of AKTi and MEKi (C) for 1 hr, 6 hr and 24 hr. The whole cell lysates were processed for western blot and probed with indicated antibodies. AKT phosphorylation levels were normalized by GAPDH and compared to NC treatment. The reactivation of AKT was calculated according to the formula: p-AKT_relative_ = p-AKT_24hr_ - p-AKT_1hr_ while the reactivation of ERK was calculated according to the formula: p-ERK_relative_= p-ERK_24hr_ - p-ERK_1hr_.

To understand the mechanisms underlying the resistance of the KRAS/BRAF mutant CRC cells to the AKTi and/or the MEKi treatments, we investigated whether AKT and ERK signaling were inhibited effectively. The AKT phosphorylation was inhibited after 1 hour AKTi treatment, but was restored within 24 hours in the five AKTi-resistant cell lines (Figure 2A). The AKT phosphorylation was restored to a less extend in the AKTi-sensitive cell line LOVO. Similarly, the ERK phosphorylation was also inhibited after 1 hour MEKi treatment, but was reactivated within 24 hours in all the six MEKi-resistant cell lines (Figure 2B). Furthermore, the combination of AKTi and MEKi could not prevent the reactivations of AKT and ERK (Figure 2C). These results suggested that the reactivation of AKT and ERK might be the reason for the resistance of the KRAS or BRAF mutant CRC cells to the AKTi and MEKi treatments.

### The increased activation of RTKs were responsible for the reactivation of AKT, but not ERK

**Figure 3:**
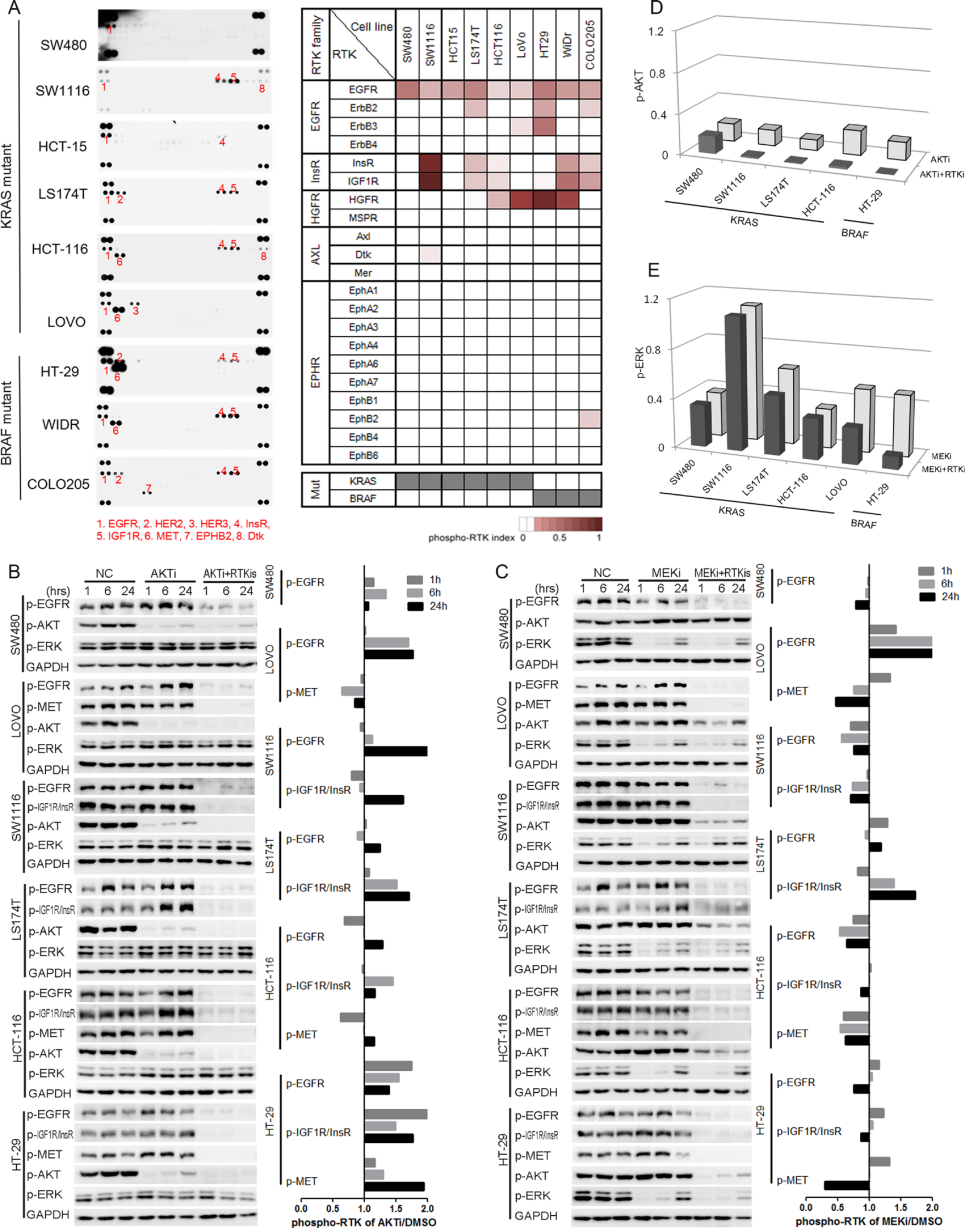
The increased activation of RTKs was responsible for the reactivation of AKT, but not ERK (**A**) RTK activation profiles of nine KRAS/BRAF mutant CRC cell lines were detected by using phospho-RTK arrays (left panel). Phospho-RTKs were numbered and illustrated below the profiles. Quantification of RTK activations was illustrated by phospho-RTK index, and shown as heat maps (right panel). Mutation status for KRAS and BRAF were shown at the bottom (gray, mutation; white, wild-type). (**B**–**C**) Cells were treated with DMSO (NC), AKTi, AKTi+RTKis combination (B), or MEKi, MEKi+RTKis combination (C) for 1 hr, 6 hr and 24 hr. The whole cell lysates were processed for western blot and probed with indicated antibodies. Line graphs indicated the quantitative changes of RTK phosphorylation under AKTi or MEKi treatment versus DMSO treatment (NC). Ratio less than 1 indicated decreased RTK phosphorylation, and ratio larger than 1 indicated increased RTK phosphorylation. (**D**) Quantification of the AKT phosphorylation changes in five AKTi-resistant CRC cell lines in (B). The AKT phosphorylation levels were normalized to GAPDH and compared to NC treatment. (**E**) Quantification of the ERK phosphorylation changes in six MEKi-resistant CRC cell lines in (C). The ERK phosphorylation levels were normalized to GAPDH and compared to NC treatment. RTKis represented individual RTK activation-based RTKi combination in each cell line: LAP for SW480, LAP+OSI for SW1116 and LS174T, LAP+OSI+JNJ for HCT-116 and HT-29, and LAP+JNJ for LOVO. The concentrations for each RTKi were as follows: LAP (L), 0.5 μM; OSI (O), 0.5 μM; JNJ (J), 0.05 μM.

We next analyzed the phosphorylation of upstream RTKs in order to understand the mechanisms of the reactivation of AKT and ERK after the AKTi and/or MEKi treatments by using the phospho-RTK arrays [4, 5, 29]. More than one RTK was activated in eight of the nine cell lines (Figure 3A). The frequently activated RTKs were EGFR family members (EGFR, HER2 and HER3), insulin receptor family members (InsR and IGF1R), and HGFR (hepatocyte growth factor receptor, also known as MET). However, the RTK phosphorylation patterns were heterogeneous among the KRAS or BRAF mutant cells (Figure 3A).

We then analyzed the RTK phosphorylation changes after the AKTi and MEKi treatments. The phosphorylation of the activated RTKs was increased in all tested cell lines after 24 hours AKTi treatment (Figure 3B). In contrast, the phosphorylation of RTKs was only induced in two cell lines (LOVO and LS174T) after 24 hours MEKi treatment and was not correlated with the reactivation of ERK (Figure 3C).

To understand the function of the activated RTKs, we treated the cells with a combination of the corresponding RTK inhibitors (RTKis) (Supplementary Figure S1) or together with the AKTi or MEKi (Figure 3B–3E). Lapatinib (LAP) was used for inhibiting EGFR family [30], OSI-906 (OSI) for Insulin Receptor family [31], and jnj38877605 (JNJ) for HGFR family [32]. The combination of RTKis and AKTi abolished the reactivation of AKT, but the combination of RTKis and MEKi did not abolish the reactivation of ERK (Figure 3B–3E). Meanwhile, the combination of MEKi and RTKis could not abolish the up-regulation of p-CRAF, which was reported to reactivate the ERK after MEKi treatment (Supplementary Figure S2) [17, 18, 33]. These data suggested that the increased phosphorylation of the RTKs by the AKTi treatment was responsible for the reactivation of AKT.

### Phospho-RTK profile-based RTKi combination treatments inhibited the growth of CRC cells by inhibiting AKT phosphorylation

**Figure 4:**
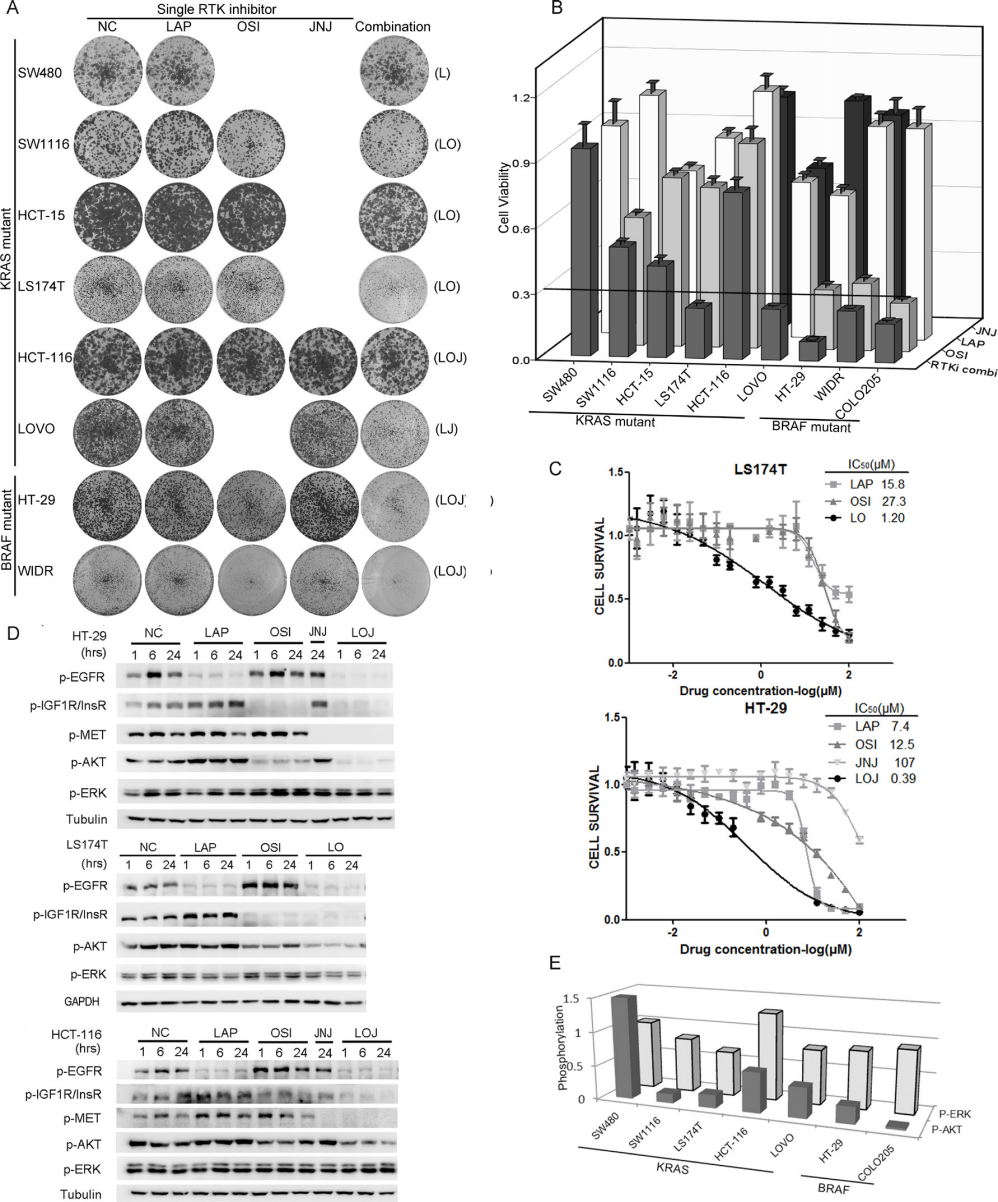
Phospho-RTK profile-based RTKi combinations partially inhibited the growth of BRAF/KRAS mutant CRC cells mainly by inhibiting AKT phosphorylation (**A**) Foci formation assay of six KRAS mutant CRC cell lines and two BRAF mutant CRC cells. Cells were treated with LAP, OSI and JNJ individually or in combination according to the specific phospho-RTK patterns in each cell line and visualized by crystal violet staining at endpoint. The combinations of RTKis were illustrated in parenthesis. The concentrations for RTKis were: LAP (L), 0.5 μM; OSI (O), 0.5 μM; JNJ (J), 0.05 μM. (**B**) Quantification of the crystal violet staining in (A). For COLO205 cells, absolute cell numbers were counted by MUSE cell analyzer. A cutoff of relative cell viability at 0.3 was drawn to define drug-sensitive and drug-resistant effects. (**C**) LS174T cells were treated with LAP, OSI or the combination of LAP and OSI (LO) with a ratio of 1:1 at various concentrations (top panel). HT-29 cells were treated with LAP, OSI, JNJ or the combination of LAP, OSI and JNJ (LOJ) with a ratio of 10:10:1 at various concentrations (bottom panel). 72 hr later, cell viability was obtained by the MTT assay. The IC_50_ values of each treatment were calculated. The IC_50_ values of RTKi combinations were represented by the concentrations of LAP in the combination. (**D**) HT-29, LS174T and HCT-116 cells were treated with DMSO (NC), 0.5 μM LAP, 0.5 μM OSI and 0.05 μM JNJ individually or in combination for 1 hr, 6 hr and 24 hr. The whole cell lysates were processed for western blot and probed with indicated antibodies. (**E**) The quantification of AKT and ERK phosphorylation under RTKi treatments in (D) and Figure S3. P-AKT and p-ERK levels at 24 hr after drug exposure were compared to the DMSO treatment.

To understand the functions of the activated RTKs for the growth of the KRAS or BRAF mutant CRC cells, the cells were treated with LAP, OSI, or JNJ individually or in combination based on the phospho-RTK patterns (Figure 4A and 4B). The concentrations we used were sufficient to completely inhibit the phosphorylation of targeted RTKs. The RTKi combinations potently inhibited the growth of the three BRAF mutant cell lines (COLO205, HT-29, and WIDR), and two of the KRAS mutant cell lines (LOVO and LS174T, more than 70% inhibition). However, four other KRAS mutant cell lines (SW480, SW1116, HCT-116, and HCT-15) were resistant to the RTKi combination treatments (less than 60% inhibition). The RTKi combinations synergistically inhibited the growth of the sensitive cells, because the IC_50_s in the combinations decreased more than 10-fold than that in the single drug treatments (Figure 4C).

We then selected three cell lines with different growth inhibition rates (HT-29, LS174T, and HCT-116), and measured the phosphorylation of RTKs and downstream effectors after drug treatments. Inhibition of a single RTK increased the phosphorylation of the remaining non-targeted RTKs. However, the RTKi combinations inhibited the phosphorylation of all targeted RTKs as well as the phosphorylation of AKT in all three cell lines (Figure 4D). We also observed strong inhibitions of AKT phosphorylation, but not that of ERK, by the RTKis treatments in six of the seven KRAS or BRAF mutant CRC cells (Figure 4E and Supplementary Figure S3). These data demonstrated that the activated RTKs supported the growth of the KRAS or BRAF mutant CRC cells through the PI3K-AKT pathway. Phospho-RTK profile-based RTKi combination treatments inhibited the AKT signaling and the growth of BRAF or KRAS mutant CRC cells.

### RTKi combinations synergized with AKTi but not MEKi to inhibit the growth of CRC cells

**Figure 5:**
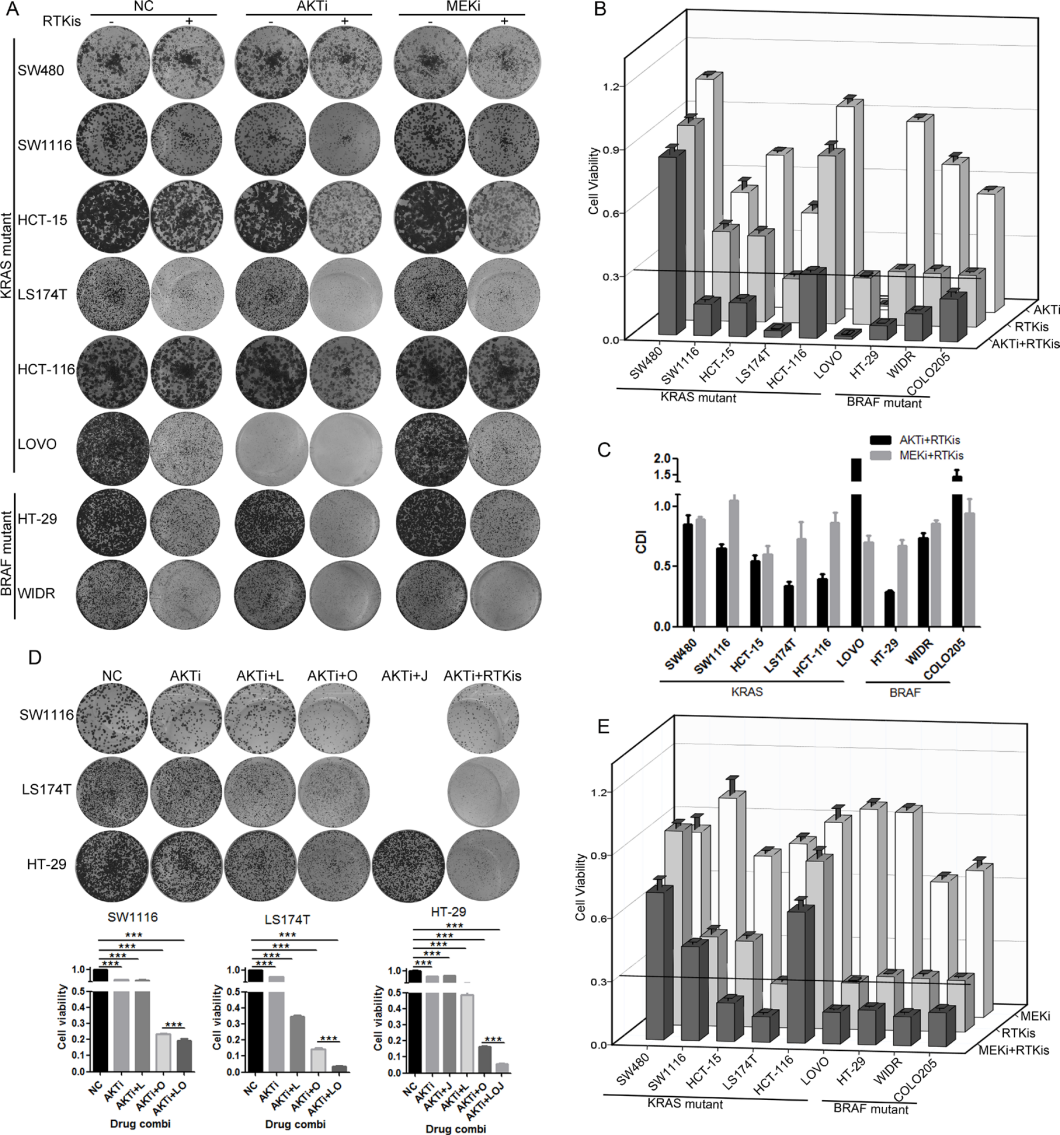
RTKi combinations synergized with AKTi but not with MEKi to inhibit the CRC growth (**A**) Foci formation assay of eight KRAS/BRAF mutant CRC cells which were treated with DMSO (NC), 0.25 μM AKTi and 0.5 μM RTKis individually or in combination, or 2.5 μM MEKi and 0.5 μM RTKis individually or in combination. Foci were visualized by crystal violet staining at endpoint. The combination and concentration of RTKis for each cell line were the same as in Figure 4A. (**B** and **E**) Quantification of the crystal violet staining in (A). For COLO205 cells, absolute cell numbers were counted by MUSE cell analyzer. A cutoff of relative cell viability at 0.3 was drawn to define drug-sensitive and drug-resistant effects. (C) The coefficient of drug interaction (CDI) values were calculated for the combination of AKTi and RTKis or the combination of MEKi and RTKis as shown in (A). CDI = 1 indicated additive effects and CDI < 1 indicated synergistic effects. (D) Foci formation assay of LS174T, HT-29 and SW1116 cells. Cells were treated with AKTi, combination of AKTi and single RTKi, or combination of AKTi and multiple RTKis. Foci were visualized by crystal violet staining at endpoint (top panel), and quantified to DMSO (NC) treatment (bottom panel). Data were represented as mean±SD. The statistical analysis was performed by one-way ANOVA with Tukey post-hoc test. ***p < 0.001.

Because the AKTi treatment increased the phosphorylation of RTKs in the KRAS or BRAF mutant CRC cells, we hypothesized that the combination of RTKis and AKTi might inhibit the cell growth more efficiently. Indeed, the growth inhibition with the combination treatments were above 70% in all cell lines except SW480 (Figure 5A and 5B). The combination of AKTi and RTKis exhibited synergistic effects in six of the nine cell lines, as indicated by the coefficient of drug interaction (CDI) (Figure 5C). Moreover, the combination of RTKis plus AKTi inhibited cell growth more potently than the single RTKi plus AKTi (Figure 5D). The combination of RTKis plus MEKi also inhibited cell growth more efficiently than the individual RTKis or MEKi (Figure 5A and 5E). However, the CDIs of the MEKi combinations were all around 1, indicating additive effects rather than synergistic effects (Figure 5C). These data demonstrated that the RTKi combinations synergized with AKTi but not MEKi to inhibit the growth of the KRAS or BRAF mutant CRC cells.

### The RTK/IRS1-mediated reactivation of AKT was responsible for the insufficient inhibition of cell growth by AKTi

**Figure 6:**
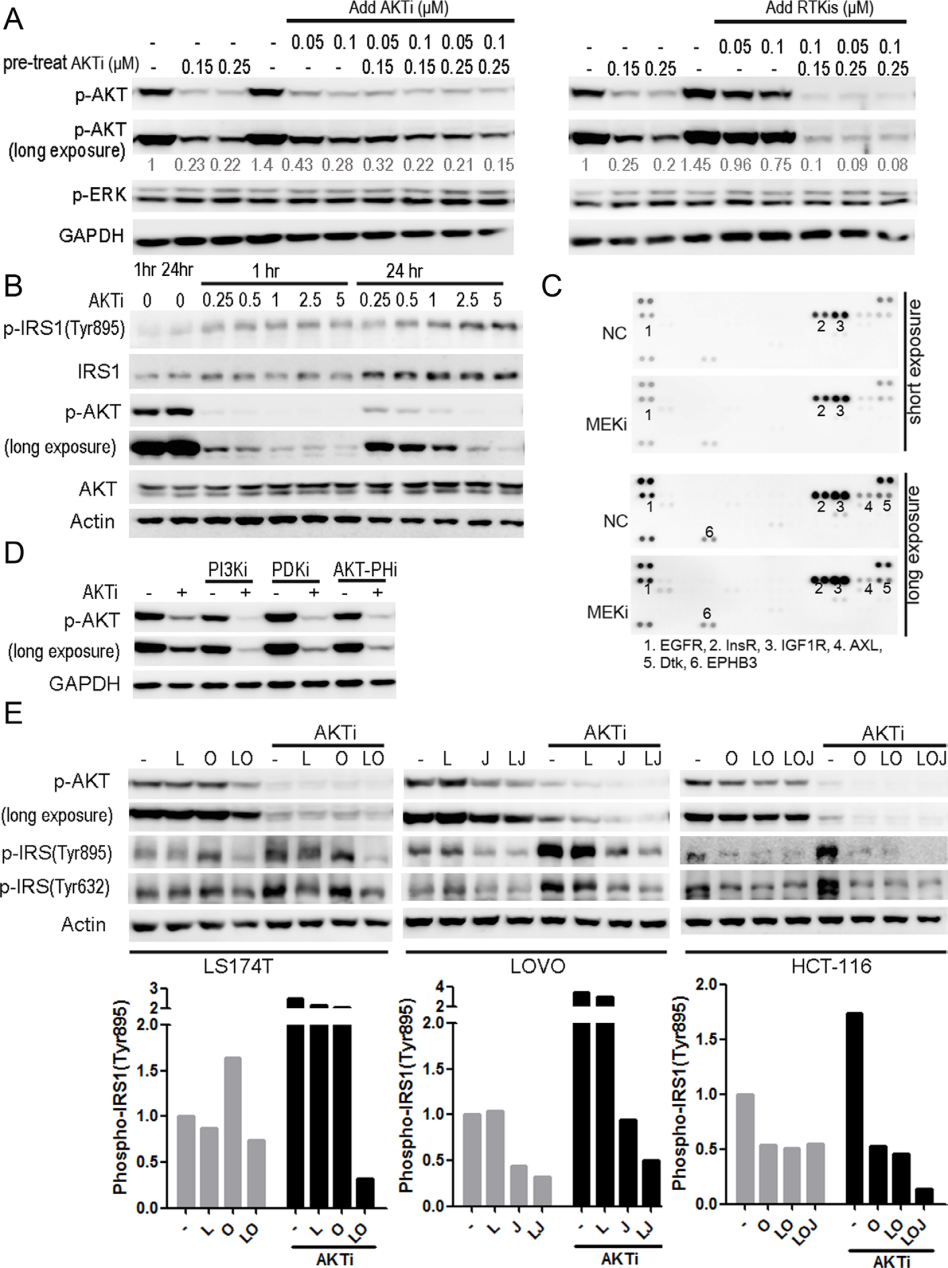
The RTK/IRS1-mediated reactivation of AKT was responsible for the insufficient inhibition of the cell growth by the AKTi (**A**) HCT-116 cells were pretreated with AKTi for 46 hr, and then treated with AKTi or RTKis (LOJ) for 2 hr. The whole cell lysates were processed for western blot and probed with indicated antibodies. Relative AKT phosphorylation levels were quantified to DMSO treatment, and illustrated as numbers below the blots. (**B**) SW1116 cells were treated with AKTi at various concentrations for 1 hr and 24 hr. The whole cell lysates were processed for western blot and probed with indicated antibodies. (**C**) Phospho-RTK arrays of SW1116 cells, which were treated with DMSO (NC) or MEKi for 24 hr. Positive dots were numbered and illustrated below the arrays. (**D**) SW1116 cells which were pretreated with 0.25 μM AKTi for 24 hr and then treated with 10 μM LY294002 (PI3Ki), 2.5 μM GSK2334470 (PDKi), or 1 μM triciribine (PIP3-AKT binding inhibitor) for 1 hr. The whole cell lysates were processed for western blot and probed with indicated antibodies. (**E**) LS174T, LOVO and HCT-116 cells were treated with single RTKi or the specific RTKis with or without AKTi for 24 hr. The whole cell lysates were processed for western blot and probed with indicated antibodies. IRS1 phosphorylation (Tyr895) was quantified and normalized to DMSO treatment (bottom panel). The drug concentrations were as follows: AKTi: 0.25 μM; LAP (L): 0.5 μM; OSI (O): 0.5 μM; JNJ (J): 0.05 μM.

The above data indicated a good correlation between the efficient inhibition of AKT phosphorylation and the inhibition of cancer cell growth. We then asked whether increasing the dose of AKTi could inhibit the AKT reactivation. The HCT-116 cells were pretreated with AKTi for 46 hours, and then treated with AKTi or RTKis for 2 hours. Additional AKTi did not inhibit the AKT reactivation but addition of low doses RTKis inhibited the reactivation of AKT (Figure 6A). Similarly, in SW1116 cells, AKT was reactivated even at the AKTi concentration of 2.5 μM, which was 10-fold of the concentration we used for a complete AKT inhibition at 1 hour (Figure 6B). Moreover, the IC_50_ of AKTi in the combination of AKTi and RTKis was nearly 100-fold lower than that of the single AKTi treatment in LS174T cells (Supplementary Table S1). The combination of AKTi and RTKis was also more cell-selective than the single AKTi treatment (Supplementary Table S1). Therefore, the combination of RTKis and AKTi was more efficient than high doses of AKTi to completely inhibit the AKT phosphorylation and the growth of the KRAS or BRAF mutant CRC cells.

In contrast, the combination of RTKis with the MEKi did not inhibit the reactivation of ERK (Figure 3C and 3E). The phosphorylation of CRAF was increased by the MEKi treatment but could not be reduced by RTKis treatment (Supplementary Figure S2). There were also no new RTKs activated concomitantly with the increased phosphorylation of CRAF after the MEKi treatment (Figure 6C), suggesting that the reactivation mechanism of ERK was different from that of AKT and the RTKs were not involved.

Because RTKs activate AKT through the PI3K-3-phosphoinositide-dependent protein kinase-1 (PDK1) pathway, we asked whether the AKT reactivation depended on PI3K and PDK1. The reactivation of AKT by AKTi treatment was inhibited by the PI3K inhibitor, the PDK1 inhibitor, or the PIP3-AKT binding inhibitor in Figure 6D, confirming that AKT was reactivated through the RTK-PI3K-AKT pathway [34–36].

Insulin receptor substrate 1 (IRS1) was reported to mediate the feedback inhibition of the RTK-PI3K-AKT pathway [37–39]. We therefore analyzed the phosphorylation of IRS1 after AKTi treatment. The Y895-phosphorylated IRS1 was increased in all of the four cell lines analyzed (Figure 6E). Further analysis demonstrated that AKTi also increased the expression of IRS1 (Figure 6B). The combinations of RTKis and AKTi blocked the induction of IRS1 phosphorylation in all the four cell lines analyzed (Figure 6E). These results suggested that a relief of a feedback inhibition of the RTK-IRS1-PI3K-AKT pathway was responsible for the reactivation of AKT after AKTi or AKTi plus MEKi treatment in the KRAS or BRAF mutant CRC cells, and the combination of RTKis and AKTi efficiently inhibited the reactivation of AKT and the cancer cell growth.

## DISCUSSION

Receptor tyrosine kinases and their downstream signaling molecules are major anti-cancer drug targets and the drugs targeting these molecules have been successful in treating certain cancers whose growth rely on the targeted molecules [10]. However, many cancers, such as colorectal cancers, contain KRAS/BRAF mutant genes and are resistant to MEKi, BRAFi, or RTKis treatments [24, 40, 41]. Therefore, we explored strategies to treat the KRAS/BRAF mutant CRC cells with various inhibitors against the key signaling molecules along the RTK signaling pathway individually or in combination. We found a reactivation of AKT and ERK after the AKTi and/or MEKi treatments as the mechanism for the cancer cell resistance to AKTi and MEKi. A relief of a feedback inhibition of the RTK-IRS1-PDK1-AKT signaling was responsible for the reactivation of AKT. The RTKi combinations synergized with AKTi to inhibit the growth of KRAS/BRAF mutant CRC cells, along with a complete inhibition of AKT phosphorylation. These data suggest a major role of the PI3K-AKT pathway in supporting the growth of the KRAS or BRAF mutant CRC cells and an effective way to inhibit the pathway.

On the contrary, the RTKs did not seem to be responsible for the reactivation of ERK after the MEKi treatment, because we did not observe increased activation of the RTKs after the treatment in most of the cell lines analyzed and the combinations of RTKis and MEKi did not block the ERK reactivation. It has been reported that CRAF was involved in the MEKi resistance in KRAS mutant cancer cells [18]. We also observed an activation of CRAF after the MEKi treatment in the CRC cells, suggesting that CRAF may be the key molecule that mediates the feedback inhibition of the RAS-RAF-MEK-ERK signaling pathway. Thus, the reactivations of AKT and ERK after the AKTi and/or MEKi treatment are mediated by distinct mechanisms, and different strategies are needed to block the reactivations and to inhibit the cancer cell growth.

The AKT kinase is an attractive drug target due to its pro-survival and pro-growth role in cancer cells [42]. However, the AKTi anti-cancer drug MK2206, when used at the dose to effectively inhibit cancer cell growth, causes several adverse effects such as rash, gastrointestinal symptoms, fatigue, and hyperglycemia, which hinder its clinical applications [43]. The combination of AKTi and RTKis may reduce the AKTi-caused toxicity by using the AKTi at a lower dose (Supplementary Table S1). Moreover, the combination of AKTi and RTKis was more selective than the single AKTi treatment among different cells.

**Figure 7:**
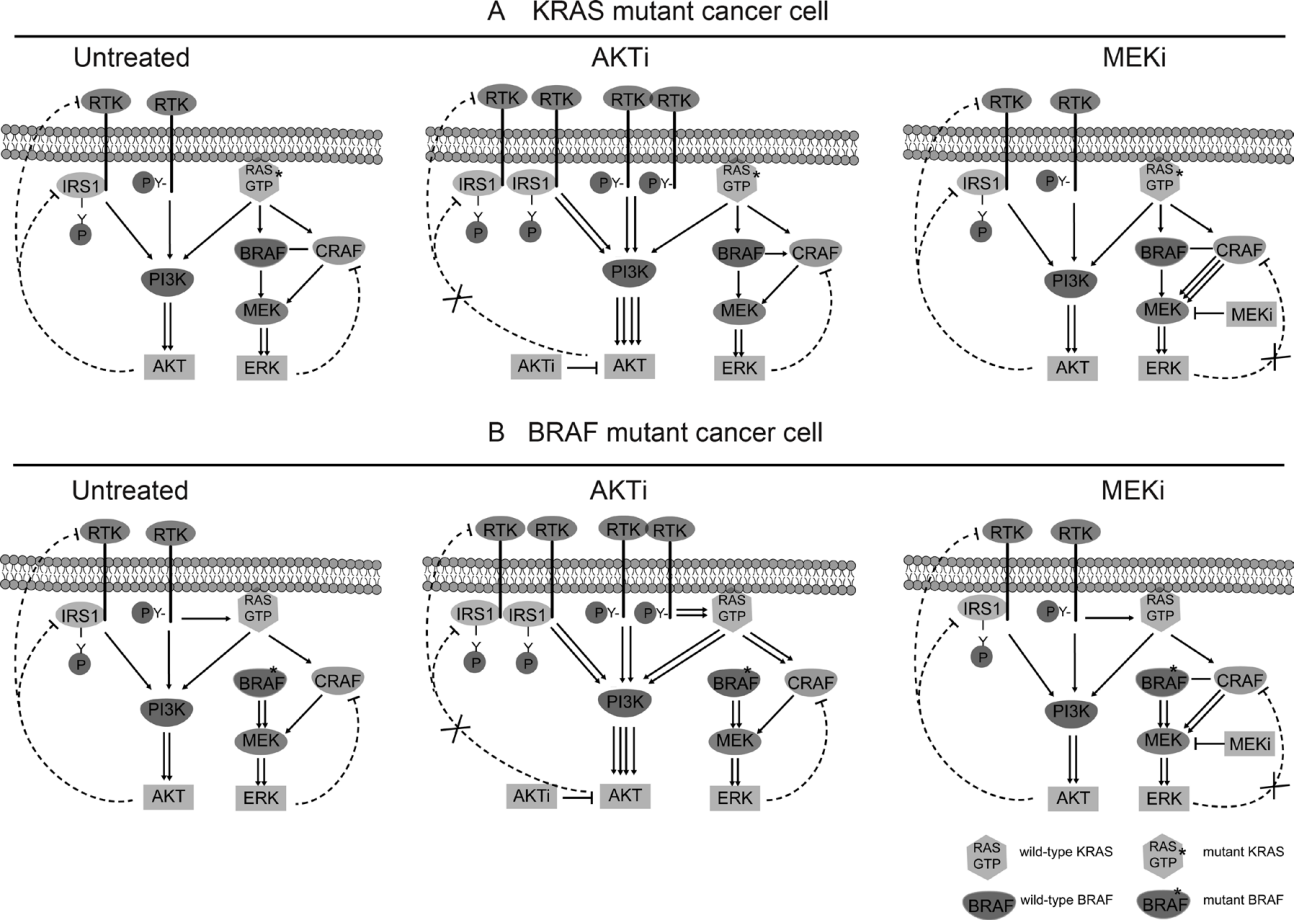
Schematic diagram of the RTK signaling pathways in the KRAS/BRAF mutant CRC cells (**A**) Regulation of AKT and ERK in KRAS mutant cancer cells. Left: Untreated cells. Middle: AKTi-treated cells. Right: MEKi-treated cells. (**B**). Regulation of AKT and ERK in BRAF mutant cancer cells. Left: Untreated cells. Middle: AKTi-treated cells. Right: MEKi-treated cells. In the KRAS mutant cells, AKT is activated by both the mutant RAS and normal RTKs, but inhibited by a negative feedback mechanism through the RTK-IRS1-PI3K pathway. ATKi inhibits the phosphorylation of AKT, but also relieves the feedback inhibition of RTK and/or IRS1, leading to reactivation of AKT. ERK on the other hand is activated mainly by the mutant RAS, while inhibited by another feedback mechanism through CRAF. MEKi inhibits the phosphorylation of ERK, but relieves the feedback inhibition of CRAF, leading to the reactivation of ERK. In the BRAF mutant cells, AKT and ERK are regulated by similar positive and negative pathways as that in the KRAS mutant cells, except that ERK is activated by the mutant BRAF instead of KRAS.

Based on these results, we propose a model to explain the functions and regulations of AKT and ERK in the KRAS or BRAF mutant CRC cells (Figure 7). In the KRAS mutant cells, AKT is activated by both the mutant RAS and normal RTKs, but inhibited by a negative feedback mechanism through the RTK-IRS1-PI3K pathway. ATKi inhibits the phosphorylation of AKT, but also relieves the feedback inhibition of RTK and/or IRS1, leading to reactivation of AKT. ERK on the other hand is activated mainly by the mutant RAS, while inhibited by another feedback mechanism through CRAF. MEKi inhibits the phosphorylation of ERK, but relieves the feedback inhibition of CRAF, leading to the reactivation of ERK. In the BRAF mutant cells, AKT and ERK are regulated by similar positive and negative pathways as that in the KRAS mutant cells, except that ERK is activated by the mutant BRAF instead of KRAS. The combination of RTKis synergized with AKTi, but not with MEKi, to completely inhibit the activation of AKT, but not that of ERK, to block the cancer cell growth.

Taken together, our data suggest a complete inhibition of AKT by a combination of RTKis, based on the RTK activation profile, and AKTi as an effective way to inhibit the growth of the KRAS or BRAF mutant CRC cells.

## MATERIALS AND METHODS

### Cell lines

SW1116 and HCT-15 cells were gifts from Prof. Meiyu Geng (Shanghai Institute of Materia Medica). All other cells were obtained from ATCC. WiDr and LS174T cells were cultured in Dulbecco's Modification of Eagle's Medium (Invitrogen) with 10% FBS. SW1116 cells were cultured in Minimum Essential Medium (Invitrogen) with 10% FBS. All other cells were cultured in RPMI 1640 Medium (Invitrogen) with 10% FBS.

### Reagents

Lapatinib, OSI-906, jnj38877605, MK2206, and U0126 were purchased from Selleck Chemicals. LY294002 and triciribine were purchased from Enzo Life Sciences. GSK2334470 was purchased from MedChem Express. The following antibodies were purchased from Cell Signaling Technology: phospho-EGFR (#3777), phospho-InsR/IGF1R (#3024), phospho-Met (#3077), phospho-Akt (#4060), phospho-Erk1/2 (#9101), phospho-CRAF (#9427), phospho-IRS1 (#3070) and IRS1 (#2382). Phospho-IRS1 (pY632) antibody (ab109543) and vinculin antibody (ab73412) were purchased from Abcam. GAPDH antibody was purchased from Shanghai Kangchen. α-tubulin antibody was purchased from Santa Cruz. And β-actin antibody was purchased from Abmart.

### Foci formation experiment

1000–5000 cells/well were seeded into 24-well plates, and treated with vehicle or indicated drugs on the following day. After 7 days, cells were fixed with methanol for 10 min and rinsed in PBS twice. Then, cells were stained with 0.1% crystal violet solution for 10 min and washed three times with PBS. The whole plates were photographed. For quantitative analysis, stained cells were dissolved with 33% acetic acid solution and the absorbance was recorded at 570 nm by SYNERGY H1 (BioTek) spectrophotometer. For COLO205, absolute cell numbers were counted by MUSE cell analyzer (Millipore). All experiments were performed in triplicate and graphically represented as mean ± SD (SPSS Statistics 19).

### MTT assay

5000 cells/well were seeded into 96-well plates, and treated with vehicle or RTKis at indicated concentrations on the following day. Three days later, MTT (5 mg/mL) were added into cells and incubated at 37°C for 3 hr. The formazan crystals were dissolved in 100 μL triplex solution (10% SDS −5% isobutanol −12 mM HCl) for 16 hr. The absorbance was measured at 595 nm by SYNERGY H1 (BioTek) spectrophotometer.

### RTK phosphorylation/activation profiling

5 mg protein lysates of the cultured cells were analyzed using phospho-RTK arrays (R&D Systems). The arrays were photographed and the phospho-RTK index was calculated according to the following formula: phospho-RTK_x_ index = (INT_x_-INT_nc_)/ (INT_ref_-INT_nc_). INT_x_ is the pixel density of RTK_x_, INT_nc_ is the pixel density of background, and INT_ref_ is the density of reference spots. Receptors were considered to be phosphorylated/activated when index values were greater than 0.1.

### Western blotting

Cells were harvested in RIPA buffer. Protein lysates were separated by SDS-PAGE, transferred to nitrocellulose membranes (GE Healthcare), probed with first antibodies, and then incubated with horseradish peroxidase-conjugated secondary antibodies. Immune complexes were detected by Immobilon^TM^ western chemiluminescence HRP substrate (Millipore) and photographed using Image Station 4000 MM PRO system (Carestream).

### Statistical analyses

Statistical analyses were performed by using GraphPad Prism 5. Data were graphically represented as mean ± SD. One-way ANOVA with Tukey post-hoc test was used in Figure 5D. Statistical significance was established for *p* < 0.001(***).

## SUPPLEMENTARY FIGURES AND TABLE


