# Supplementary material for: Concomitant inhibition of receptor tyrosine kinases and downstream AKT synergistically inhibited growth of KRAS/BRAF mutant colorectal cancer cells
Source: Oncotarget. 2016 Dec 17;8(3):5003–15. doi: 10.18632/oncotarget.14009 (PMC5354887; doi:10.18632/oncotarget.14009)
Supplement: Supplementary file 1 [file oncotarget-08-5003-s001.pdf]

Concomitant inhibition of receptor tyrosine kinases and downstream AKT synergistically inhibited growth of KRAS/BRAF mutant colorectal cancer cells

Supplementary Materials

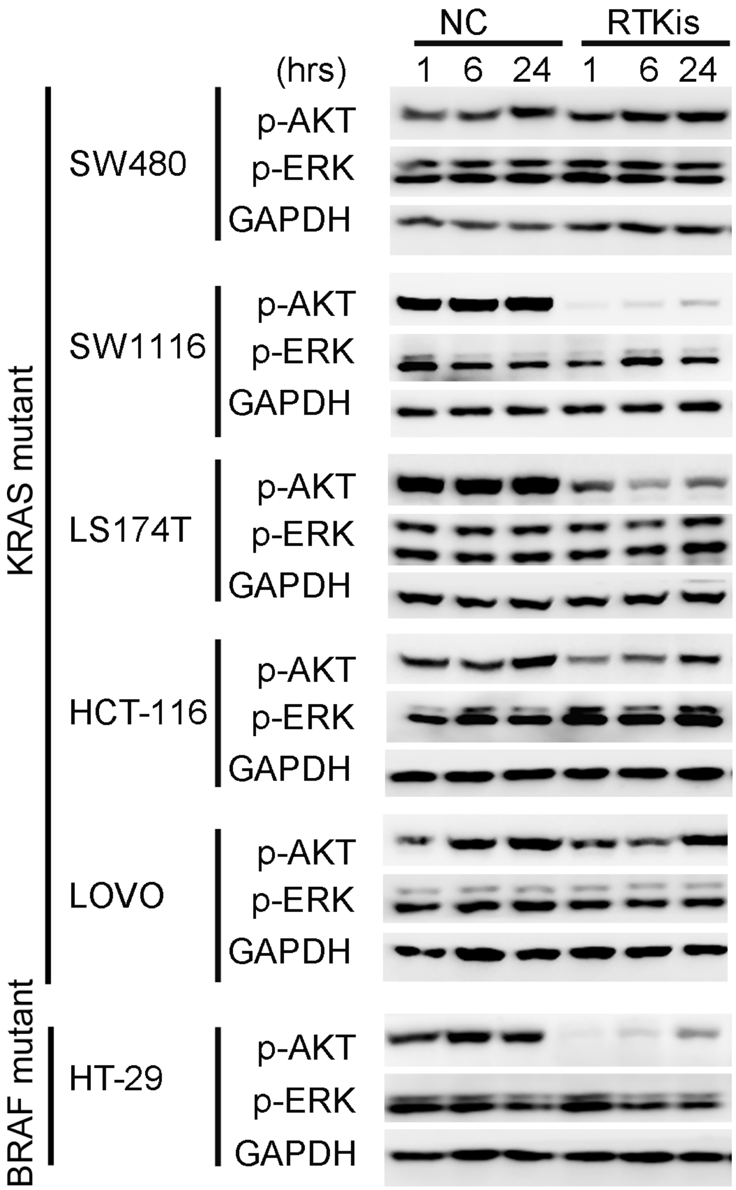

Supplementary Figure S1: RTKis combination inhibited the phosphorylation of AKT but not ERK in the KRAS or BRAF mutant CRC cells. Cells were treated with DMSO (NC) or RTKis combination for 1 hr, 6 hr, or 24 hr. The whole cell lysates were processed for western blot analyses and probed with indicated antibodies. RTKis represented different individual or combinations of RTKis according to the RTK activation profile in each cell line: LAP for SW480, LAP+OSI for SW1116 and LS174T, LAP+OSI+JNJ for HCT-116 and HT-29, and LAP+JNJ for LOVO. The concentrations for each RTKi were as follows: LAP, 0.5 µM; OSI, 0.5 µM; JNJ, 0.05 µM. Anti-GAPDH antibody was used as a protein equal loading control.

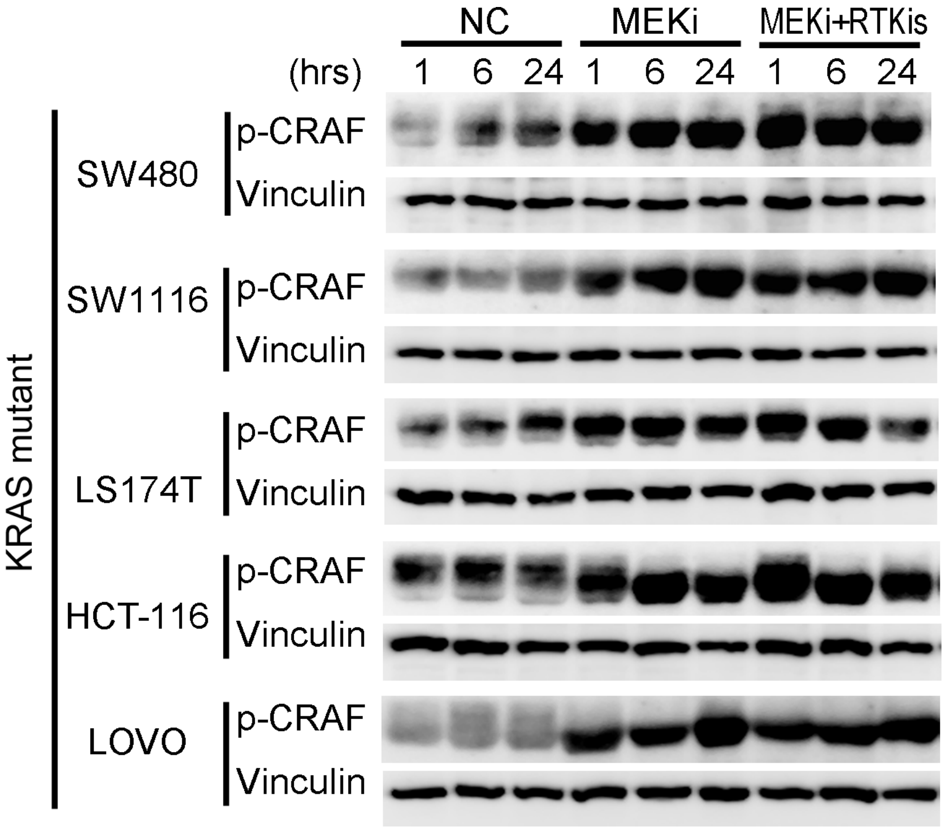

Supplementary Figure S2: RTKis combination could not inhibit the MEKi-induced CRAF phosphorylation. Cells were treated with DMSO (NC), MEKi, or the combination of MEKi and RTKis for 1 hr, 6 hr, or 24 hr. The whole cell lysates were processed for western blot analyses and probed with indicated antibodies. RTKis represented different individual or combinations of RTKis according to the RTK activation profile in each cell line: LAP for SW480, LAP+OSI for SW1116 and LS174T, LAP+OSI+JNJ for HCT-116, and LAP+JNJ for LOVO. The concentrations for each RTKi were as follows: LAP, 0.5 µM; OSI, 0.5 µM; JNJ, 0.05 µM. Anti-Vinculin antibody was used as a protein equal loading control.

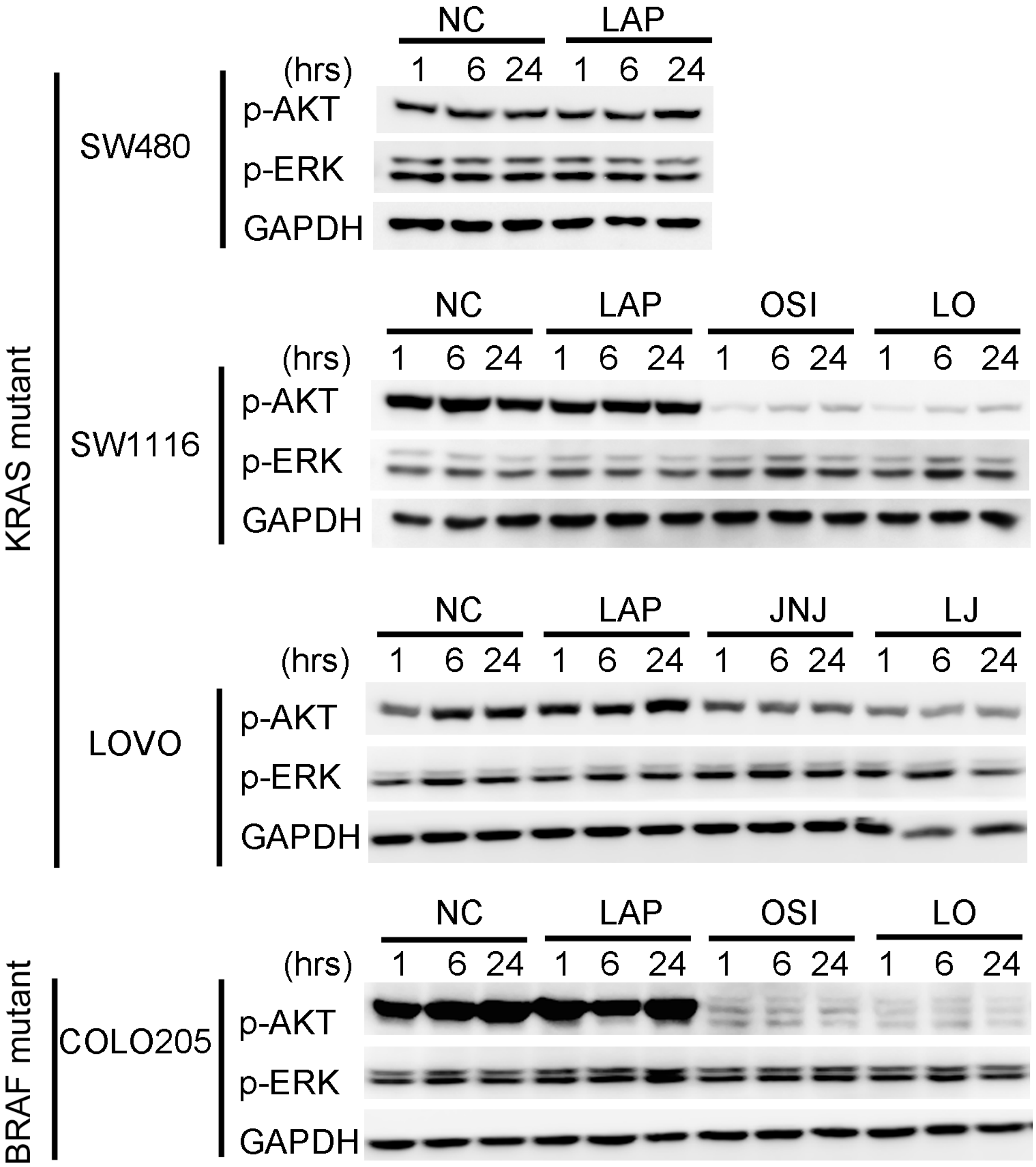

Supplementary Figure S3: The phosphorylation of AKT but not ERK was inhibited by single or combinations of RTKis. Cells were treated with DMSO (NC), 0.5 µM LAP, 0.5 µM OSI, or 0.05 µM JNJ individually or in combinations for 1 hr, 6 hr or 24 hr according to the specific RTK phosphorylation pattern in each cell line. The whole cell lysates were processed for western blot analyses and probed with indicated antibodies. Anti-GAPDH antibody was used as a protein equal loading control. LO stands for LAP+OSI; LJ stands for LAP+JNJ.

Supplementary Table S1: Growth inhibition of cancer cells by different inihibitors or inhibitor combination

Treatment	L		O		AKTi		AKTi(+L+O)	
**Cell line**	**IC_{50}**	**r squared**	**IC_{50}**	**r squared**	**IC_{50}**	**r squared**	**IC_{50}**	**r squared**
LS174T	9.4	0.97	25.4	0.978	14.2	0.984	0.15	0.994
SW480	7.9	0.979	45	0.959	14.3	0.982	1.95	0.958
HCT-116	6.7	0.977	13.6	0.937	17.3	0.96	4.3	0.986

Notes: The combinaiton of L+O was used according to the RTK phosphorylation profile of LS174T cells. The IC_{50} of the combination of AKTi(+L+O) was the IC_{50} of AKTi in the combination. L: lapatinib; O: OSI-906; AKTi: MK2206; the ratio of AKTi:L:O is 1:2:2.
